# METTL3 Intensifies the Progress of Oral Squamous Cell Carcinoma via Modulating the m6A Amount of PRMT5 and PD-L1

**DOI:** 10.1155/2021/6149558

**Published:** 2021-08-23

**Authors:** Yilong Ai, Shiwei Liu, Hailing Luo, Siyuan Wu, Haigang Wei, Zhe Tang, Xia Li, Xiaozhi Lv, Chen Zou

**Affiliations:** ^1^Foshan Stomatological Hospital, School of Stomatology and Medicine, Foshan University, Foshan, Guangdong, China; ^2^Department of Stomatology, Foshan First People's Hospital, Foshan, Guangdong, China; ^3^Department of Oral & Maxillofacial Surgery, Nanfang Hospital, Southern Medical University, Guangzhou, Guangdong, China

## Abstract

N6-Methyladenosine (m6A) modification is one of the commonest chemical modifications in eukaryotic mRNAs, which has essential effects on mRNA translation, splicing, and stability. Currently, there is a rising concern on the regulatory role of m6A in tumorigenesis. As a known component in the m6A methyltransferase complex, METTL3 (methyltransferase-like 3) plays an essential role in m6A methylation. Till now, the functions of METTL3 in oral squamous cell carcinoma (OSCC) and its relative mechanism remain to be explored. In this research, through the GEPIA database, we found that high METTL3 expression has a correlation with poor prognosis of squamous cell carcinoma of head and neck. qRT-PCR displayed that METTL3 was highly expressed in OSCC cells. Functionally, METTL3 knockdown reduced the invasion, migration, and proliferation competence of OSCC cells and attenuated the activation of CD8+ T cells. In contrast, METTL3 overexpression resulted in opposite results. GEPIA, UALCAN, and SRAMP databases, PCR, western blot, and m6A RNA methylation assay confirmed the m6A modification of PRMT5 and PD-L1 mediated by METTL3. In conclusion, our results displayed that METTL3 intensified the metastasis and proliferation of OSCC by modulating the m6A amounts of PRMT5 and PD-L1, suggesting that METTL3 may be a therapeutic target for OSCC patients.

## 1. Introduction

Oral squamous cell carcinoma (OSCC) is the commonest head and neck malignancy and responsible for approximately 90% of malignant epithelial tumors in the oral and maxillofacial region [1–4]. Over the years, comprehensive treatments, which include radiotherapy, chemotherapy, and surgical treatment, have made progress, but poor prognosis remains in OSCC patients, and the 5-year survival rate is roughly 60 [5–9]. Hence, a key to invent effective targeted therapies and improve patient prognosis is uncovering the molecular mechanisms underneath OSCC invasion and metastasis. Recently, it was identified that many genetic and epigenetic changes have a correlation with the progress of OSCC. However, there are not many studies focusing on the m6A methylation modification and OSCC.

N6-Methyladenosine (m6A) is an important RNA modification, and it is a reversible behavior catalyzed by methyltransferase complexes (m6A “writers”), demethylated transferases (m6A “erasers”), and binding proteins (m6A “readers”) [10, 11]. Methyltransferase complexes include METTL14, KIAA1429, WTAP, and METTL3 [12, 13]. m6A exerts a crucial regulatory influence in biological functions and is involved in various pathological and physiological processes [14]. The amount of m6A will influence the RNA metabolism such as the processing or translation of the modified RNA and degradation of mRNA. Its aberrant variation will result in attenuating the modulation of gene expression and intensifying the emergence of abnormal cell behavior [15, 16]. m6A enzyme system aberrant expression can be an essential influence attenuating the enrichment of m6A, consequently attenuating the expression of oncogenes or tumor suppressor genes in diverse cancers [17–19]. Countless studies have demonstrated that abnormal m6A RNA methylation exerts an essential influence in the pathogenesis of many human disorders including OSCC [14, 20, 21].

METTL3 is one of the most important proteins in the m6A methyltransferase complex, which is mainly responsible for catalyzing the m6A modification of RNA molecules on N6-methyladenine [22, 23]. The deletion or abnormal expression of METTL3 will affect the level of m6A of intracellular RNA and then affect the degradation and translation of mRNA and the generation of microRNA, which may lead to the occurrence of human diseases [24, 25]. Therefore, intracellular stabilization of METTL3 is important for the regulation of RNA m6A amounts. METTL3 is highly expressed in most human cancers, and we found through the GEPEIA database (http://gepia.cancer-pku.cn/index.html) that METTL3 has a close relation with the prognosis of squamous cell carcinoma of head and neck. The low expression of METTL3 predicts a high free survival of disease of head and neck squamous cell carcinoma. OSCC is the commonest head and neck malignancy. Therefore, this research probed the role and clinical effect of METTL3 in OSCC.

In this study, we evaluated the function of METTL3 in OSCC and probed into the mechanism of METTL3's involvement in OSCC.

## 2. Materials and Methods

### 2.1. Cell Culture and Transfection

We purchased oral squamous cell lines SCC-4, CAL-27, SCC-9, and SCC-25 from ATCC (Manassas VA, USA). We obtained healthy oral keratinocytes (HOK) from the cell bank of the Chinese Academy of Sciences (Shanghai, China). We cultured SCC-4, CAL-27, SCC-9, and SCC-25 cells in DMEM/F12, which is supplemented with 10% fetal bovine serum (FBS) and 1% penicillin-streptomycin. We cultured HOK cells with RPMI 1640 medium, which contains 15% FBS and 1% antibiotics. We cultured all of the cells at 37°C in a 5% CO_2_ incubator.

To knock down METTL3 and PRMT5, lentivirus constructs were generated. The OSCC cells were infected stably with METTL3-knockdown lentivirus (sh-METTL3#1, sh-METTL3#2), PRMT5-knockdown lentivirus (sh-PRMT5), and pLKDCMV-G&PR-U6 negative control vectors (sh-NC), following the manufacturer's instructions (OBiO Technology, China). The OSCC cells were stably transfected with pLenti-CMV-3FLAG negative control vectors (vector) and overexpression lentivirus (METTL3 OE). Cells were selected for 14 days. For METTL3 knockdown, the construct sh-METTL3#1 with the highest efficiency was utilized for a mechanism study, which was named after sh-METTL3.

### 2.2. Quantitative Real-Time Polymerase Chain Reaction (qRT-PCR)

As per the product specification, we implemented total RNA extraction via the TRIzol reagent offered by Life Technologies (Shanghai, China), and a NanoDrop 2000 spectrophotometer offered by Thermo Fisher Scientific (Shanghai, China) was utilized for the measurement of RNA concentration. Later, we carried out reverse transcription via the PrimeScript RT kit from Takara (Dalian, China). Lastly, qRT-PCR was executed by SYBR Premix DimerEraser (Takara) and StepOnePlus Real-Time PCR System offered by Applied Biosystems (Shanghai, China), with U6 and GAPDH as internal references. Primers are displayed below:

METTL3: F: 5′-GAGATATGCTCTTAACCACCCG-3′ and R: 5′-GCTGCCCAATCCATCCAA-3′

PRMT5: F 5′-CTGTCTTCCATCCGCGTTTCA-3′ and R: 5′-GCAGTAGGTCTGATCGTGTCTG-3′

PD-L1: F 5′-GGTGAGGATGGTTCTACACAG-3′ and R: 5′-GAGAACTGCATGAGGTTGC-3′

### 2.3. m6A RNA Methylation

We performed total RNA extraction via TRIzol (Thermo Fisher Scientific, China) as per the manufacturer's instructions. We used EpiQuik m6A RNA Methylation Quantification Kit (colorimetric; P-9005, EpiGentek, USA) for determining the relative content of m6A in total RNAs as per the manufacturer's instructions, and we analyzed the m6A amount colorimetrically on the basis of an optical density (OD) at 450 nm.

### 2.4. Cell Counting Kit-8 (CCK8) Experiment

We seeded about 2 × 10^3^ OSCC cells per well into the 96-well plates and cultured them for described times. Then, as per the manufacturer's protocol, we determined proliferation through the Cell Counting Kit-8 (CCK8, Dojindo, Tokyo, Japan).

### 2.5. EdU Experiment

The effects of METTL3 and PRMT5 on OSCC cell proliferation were evaluated via Click-iT® EdU Imaging Kits offered by Invitrogen as per the product specification. We cultured cells in ninety-six-well plates (8 × 10^3^/well) and incubated them using 10 *μ*L of EdU reagent for three hours. At room temperature, we fixed them with 4% formaldehyde for 20 min, and with PBS, formaldehyde was washed away. Then, cells underwent twenty minutes of incubation at room temperature utilizing 0.5% Triton X-100 from Sigma (Shanghai, China). Afterwards, we added 1 mL of Hoechst 33342 nuclear staining solution (Sigma) to each well for twenty-five minutes of incubation at room temperature in a dark place. Afterwards, we removed the staining solution by washing with PBS thrice. Ultimately, we photographed and counted the stained cells via a fluorescence microscope (CKX41-F32FL), which is from Olympus (Beijing, China).

### 2.6. Wound Healing Assay

In 6-well plates, we seeded OSCC cells and cultured them until >95% confluence. Then, using a sterile plastic tip, we gently scratched the cell layer through the central axis. We washed loose cells away and replaced the media with serum-free media. For each condition and time point (0, 24 h), we implemented quantification of cell motility through measuring the distance between the invading fronts of cells in four randomly selected microscopic fields (×100).

### 2.7. Cell Invasion Assay

To evaluate cell invasion potential, we used transwell inserts (8 *μ*m pore size; Costar, Cambridge, MA). We seeded about 2 × 10^4^ cancer cells in serum-free medium into the upper chamber coated with Matrigel (BD Biosciences, San Jose, CA). In the bottom chamber, we filled seven hundred and fifty microlitres of complete DMEM. We removed the cells in the upper chamber after culturing for 24 h and fixed the cells in the bottom chamber with 4% paraformaldehyde and stained them with 0.1% crystal violet. Via a light microscope, we counted the invaded cell numbers.

### 2.8. Western Blot Experiment

Subsequent to digestion and resuspension of OSCC cells after transfection, we inoculated the cells in six-well plates (1 × 10^6^/well) for four hours, and the protein expression amount of METTL3, PRMT5, IL-10, IL-4, IFN-*γ*, and PD-L1 was evaluated. Afterwards, we used RIPA lysate to isolate the total protein from cells and determined its total amount. Afterwards, we took 100 *μ*g proteins from each group for electrophoresis and transferred them onto PVDF membranes. For hypothermal incubation overnight, we utilized the primary antibody, following one hour of sealing at room temperature. We added secondary antibodies labeled with horseradish peroxide for one hour of incubation the next day, followed by color development utilizing a coloring reagent and photographing.

### 2.9. Tumor Xenograft Implantation in Nude Mice

Six-week-old nude mice were randomly divided into two groups (three mice per group) and cultured with continuous access to sterile food and water in pathogen-free sterile conditions. To establish the OSCC xenograft model, we subcutaneously injected 5 × 10^6^ SCC-9 cells stably transfected with METTL3 shRNA or sh-NC vectors into nude mice. Tumor growth was monitored weekly and calculated as follows: volume = (length) × (width)^2^/2. The study was approved by the Ethics Committee of Foshan Stomatological Hospital, School of Stomatology and Medicine, Foshan University, and experiments were performed following the NIH guidelines on animal welfare.

### 2.10. Data Analysis

We implemented all assays thrice separately and analyzed data via SPSS 20.0 software. Data were displayed as mean ± SD. Independent two-sample *t*-test was adopted for intergroup comparison, and the Pearson method was utilized for correlation analysis. The variation has statistical significance in the case of *p* < 0.05.

## 3. Results

### 3.1. METTL3 Is Elevated in OSCC Cell Lines

**Figure 1:**
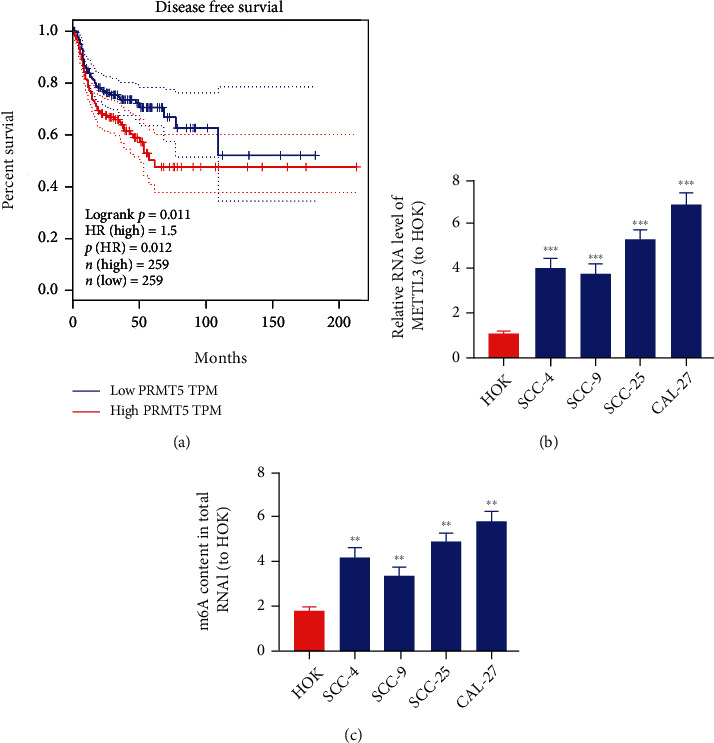
METTL3 is elevated in OSCC cell lines. (a) The effect of METTL3 amount on squamous cell carcinoma of head and neck patient survival, from the GEPIA database (http://gepia.cancer-pku.cn/index.html). (b) METTL3 expression level in OSCC cell lines (SCC-4, SCC-9, SCC-25, and CAL-27) and healthy oral keratinocyte (HOK) cells. (c) Detection of m6A amount of METTL3 in OSCC cells via methylation. ^∗∗^*p* < 0.01, ^∗∗∗^*p* < 0.001.

METTL3 was found to be closely related to the prognosis of squamous cell carcinoma of head and neck through the GEPIA database (http://gepia.cancer-pku.cn/index.html) (Figure 1(a)). The overall survival in high-expression METTL3 was remarkably shorter than that in low-expression METTL3. As the most common head and neck malignancy, OSCC is responsible for roughly 90% of malignant epithelial tumors in the oral and maxillofacial region. And METTL3 is reported to be overexpressed in most human cancers. Therefore, we want to probe into the function of METTL3 in the OSCC and its mechanism. METTL3 mRNA amount was also identified to be expressed higher in OSCC cells, in comparison to that in healthy oral keratinocyte (HOK) cells (Figure 1(b)). Then, we analyzed the amount of total methylated RNA (m6A) in OSCC cell lines. The amount of m6A was remarkably increased in OSCC cell lines, which is displayed in Figure 1(c).

### 3.2. METTL3 Intensified OSCC Cell Proliferation and Metastasis and Suppressed CD8+ T Cell Activation

**Figure 2:**
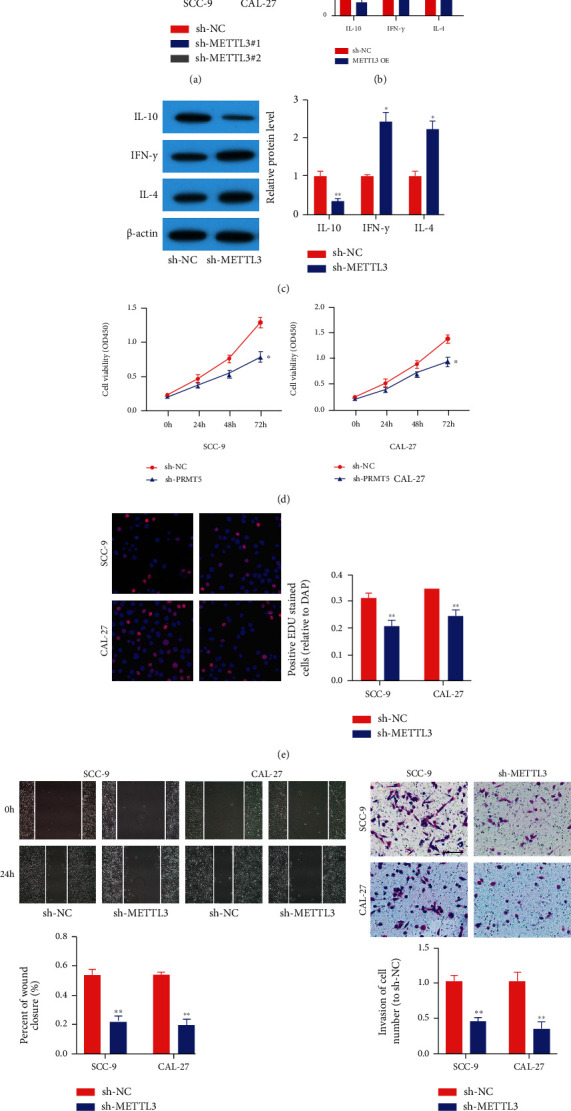
sh-METTL3 suppressed OSCC cell metastasis and proliferation and intensified CD8+ T cell activation. (a) qRT-PCR results displayed that METTL3 amount was decreased after transfection with sh-METTL3#1 and sh-METTL3#2. The construct sh-METTL3#1 with the highest efficiency was utilized for a mechanism study, which was named as sh-METTL3. (b, c) sh-METTL3 intensified CD8+ T cell activation. (d, e) CCK8 and EdU assays displayed that sh-METTL3 attenuated OSCC cell proliferation. (f) Wound healing assay displayed that sh-METTL3 suppressed the competence of OSCC cell migration. (g) Transwell assays displayed that sh-METTL3 suppressed the competence of OSCC cell invasion. ^∗^*p* < 0.05, ^∗∗^*p* < 0.01, and ^∗∗∗^*p* < 0.001.

For discovering the role of METTL3 in OSCC, we evaluated the effects of METTL3 on CD8+ T cell activation and tumor cell phenotypes. We transfected CAL-27 and SCC-9 cell lines with sh-METTL3#1 and sh-METTL3#2 to knock down the expression amount of METTL3 (Figure 2(a)). We used the construct sh-METTL3#1 with the highest efficiency for the mechanism study, which was named as sh-METTL3. We utilized ELISA and western blot to determine the amounts of antitumor cytokines IL-4 and IFN-*γ* as well as protumor cytokine IL-10 (Figures 2(b) and 2(c)). We found that sh-METTL3 transfection exhibited a decreased amount of IL-10 yet increased amounts of IL-4 and IFN-*γ*. This suggests that sh-METTL3 intensifies CD8+ T cell activation. Then, the CCK-8 and EdU assays displayed that proliferation of OSCC cells was remarkably attenuated in the sh-METTL3 group, compared to the sh-NC group (Figures 2(d) and 2(e)). Moreover, METTL3 silencing remarkably attenuated the migratory and invasive capacities of SCC-9 and CAL-27 cells (Figures 2(f) and 2(g)).

**Figure 3:**
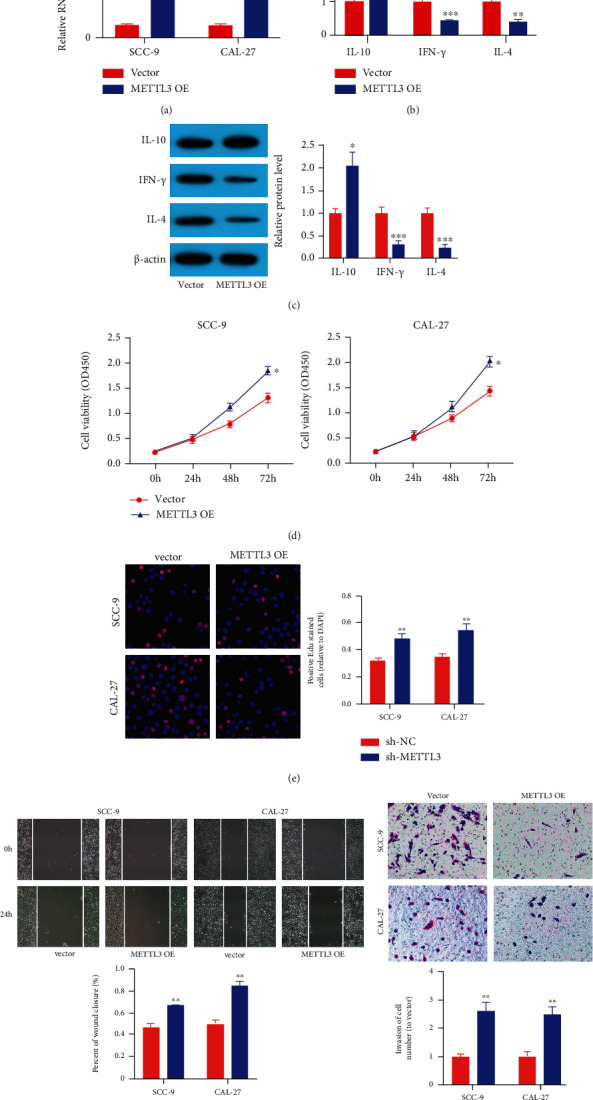
METTL3 intensified OSCC cell proliferation and metastasis and suppressed CD8+ T cell activation. (a) qRT-PCR results displayed that METTL3 amount was higher after transfection with METTL3 overexpression vectors. (b, c) METTL3 overexpression suppressed CD8+ T cell activation. (d, e) CCK8 and EdU assays displayed that METTL3 overexpression accelerated OSCC cell proliferation. (f) Wound healing assay displayed that METTL3 overexpression accelerated the competence of OSCC cell migration. (g) Transwell assays displayed that METTL3 overexpression intensified the competence of OSCC cell invasion. ^∗^*p* < 0.05, ^∗∗^*p* < 0.01, and ^∗∗∗^*p* < 0.001.

We then transfected CAL-27 and SCC-9 cell lines with METTL3 overexpression vectors (Figure 3(a)). The upmodulated cell line was named as METTL3 OE; however, the matched control cell line was named after the vector. We found that METTL3 OE transfection exhibited an increased amount of IL-10 yet decreased amounts of IL-4 and IFN-*γ* (Figures 3(b) and 3(c)). This indicates that METTL3 overexpression inhibits CD8+ T cell activation. The CCK-8 and EdU assays displayed that proliferation of CAL-27 and SCC-9 cells was remarkably intensified in the METTL3 OE group, compared to the vector group (Figures 3(d) and 3(e)). Furthermore, METTL3 OE markedly accelerated the invasion and migration of SCC-9 and CAL-27 cells (Figures 3(f) and 3(g)). These results displayed that METTL3 could intensify the cell metastasis and proliferation of OSCC in vitro and suppress CD8+ T cell activation.

### 3.3. METTL3 shRNA Limited the Growth of OSCC In Vivo

**Figure 4:**
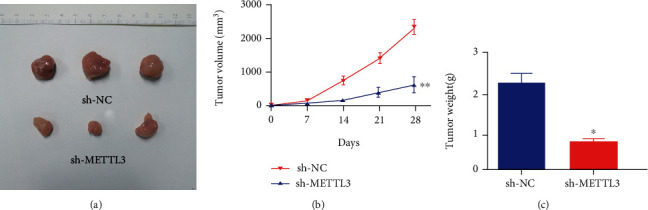
METTL3 shRNA limited the growth of OSCC in vivo. (a) Representative images of tumors from indicated xenografts. (b) Tumor volume and (c) weight data of indicated xenografts. ^∗^*p* < 0.05, ^∗∗^*p* < 0.01.

We generated xenograft models to verify the findings in this study. SCC-9 cells transfected with METTL3 shRNA were subcutaneously injected into nude mice. The results showed that METTL3 shRNA greatly limited tumor proliferation in vivo (Figures 4(a)–4(c)).

### 3.4. PRMT5 and PD-L1 Might Be the Potential Targets Modulated by METTL3 in OSCC

**Figure 5:**
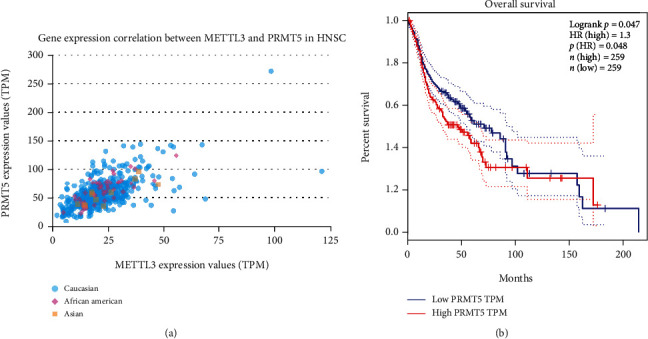
PRMT5 is elevated in OSCC and has association with poor prognosis. (a) METTL3 correlation analysis on the UALCAN database. (b) The relationship between PRMT5 amount and squamous cell carcinoma of head and neck patient survival, from the GEPIA database. ^∗∗∗^*p* < 0.001.

With the UALCAN database (http://ualcan.path.uab.edu/analysis.html), we found that multiple genes are positively associated with METTL3 expression. The Pearson test displayed that the top ten genes in a correlation include SUPT16H, PARP2, APEX1, ACIN1, METT11D1, TMEM55B, PRMT5 (Figure 5(a)), GMPR2, RAB2B, and OXA1L. Next, via the GEPIA database (http://gepia.cancer-pku.cn/index.html), we found that the higher the PRMT5 expression was, the worse the prognosis was (Figure 5(b)).

**Figure 6:**
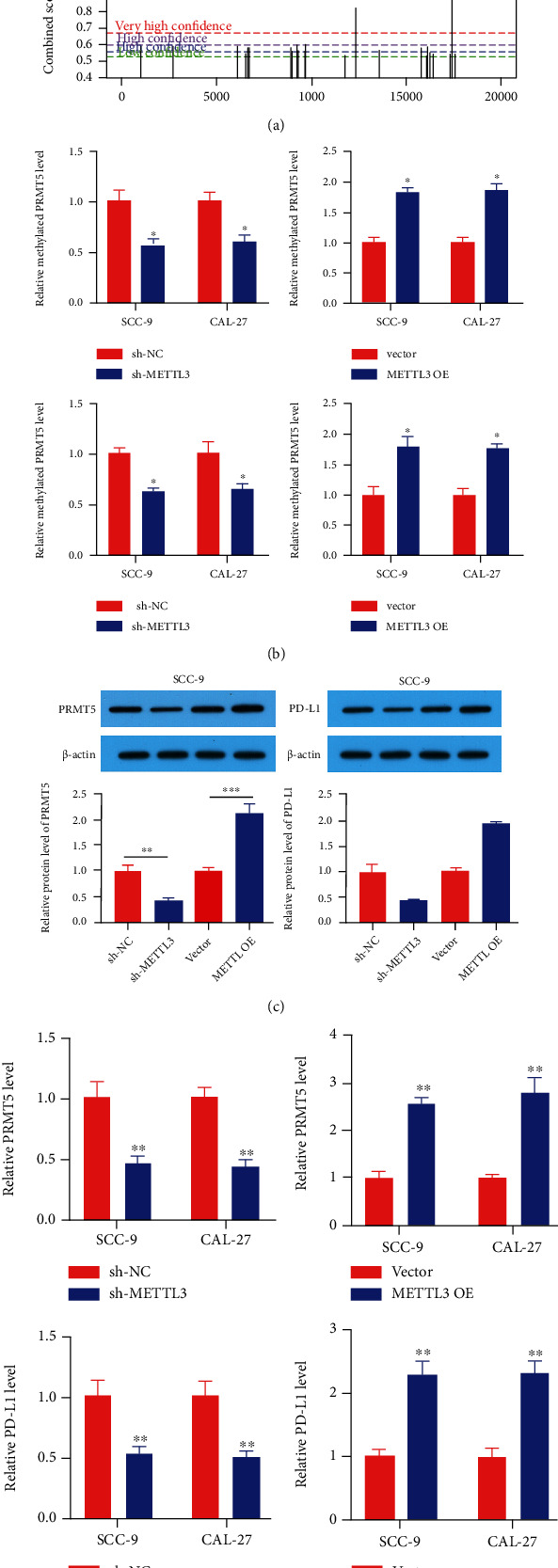
PRMT5 and PD-L1 might be the potential targets modulated by METTL3 in OSCC. (a) Bioinformatics predicted that PRMT5 and PD-L1 could produce m6A methylation at multiple sites. (b) Detection of m6A methylated amount of METTL3 and PD-L1 in OSCC cells after transfection. (c, d) The protein and RNA level of OSCC and PD-L1 cells after transfection. ^∗^*p* < 0.05, ^∗∗^*p* < 0.01.

Next, we found by the SRAMP database (http://www.cuilab.cn/sramp) that the retrieval of PRMT5 and PD-L1 can occur at multiple sites of m6A methylation (Figure 6(a)). Through methylation detection, we found that the m6A amounts of PRMT5 and PD-L1 in OSCC cells transfected with sh-METTL3 were remarkably lower than those in the sh-NC group, while the m6A amounts of PRMT5 and PD-L1 in OSCC cells transfected with METTL3 OE were remarkably higher than those in the vector group (Figure 6(b)). As shown in Figures 6(c) and 6(d), the expression amounts of PRMT5 and PD-L1 were remarkably decreased in sh-METTL3-transfected OSCC cells, while the expression amounts of PRMT5 and PD-L1 were remarkably increased in METTL3 OE-transfected OSCC cells.

### 3.5. Silencing PRMT5 and PD-L1 Attenuated the Occurrence and Development of OSCC

**Figure 7:**
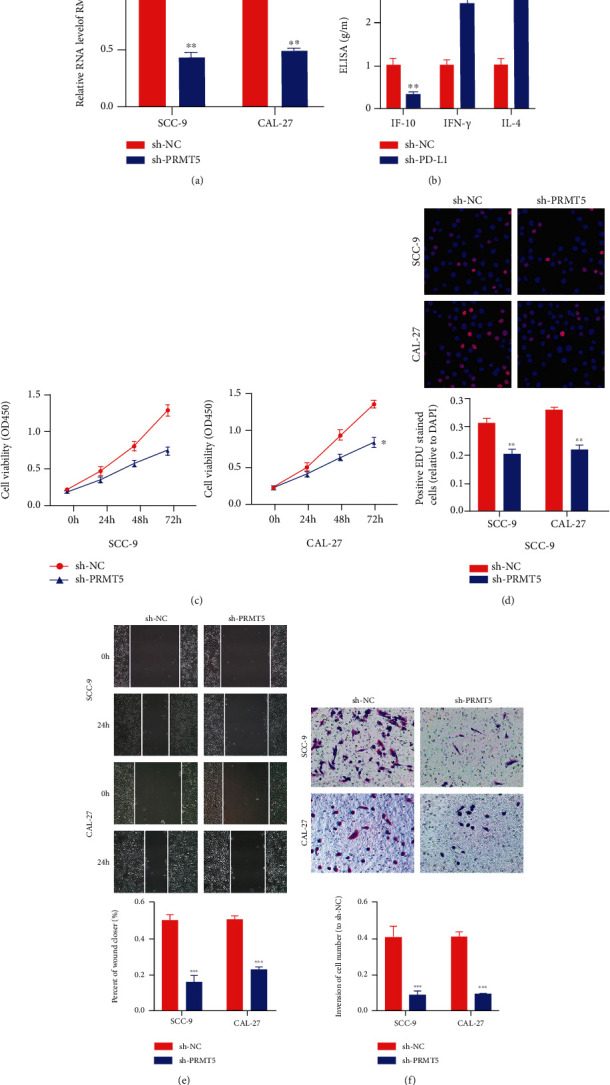
Silencing PRMT5 and PD-L1 attenuated the occurrence and development of OSCC. (a) The amount of PRMT5 in SCC-9 and CAL-27 cells after being transfected with sh-PRMT5. (b) sh-PD-L1 intensified the immune activity of T cells. (c, d) CCK8 and EdU assays displayed that sh-PRMT5 attenuated OSCC cell proliferation. (e) Wound healing assay displayed that sh-PRMT5 suppressed the competence of OSCC cell migration. (f) Transwell assays displayed that sh-METTL3 attenuated the competence of OSCC cell invasion. ^∗^*p* < 0.05, ^∗∗^*p* < 0.01, and ^∗∗∗^*p* < 0.001.

To verify the role of PRMT5 and PD-L1, we downmodulated the expression amounts of PRMT5 and PD-L1 with shRNA (Figure 7(a)). We found that sh-PD-L1 attenuated IL-10 secretion and intensified IL-4 and IFN-*γ* secretion (Figure 7(b)), indicating that sh-PD-L1 promotes CD8+ T cell activation. Then, the CCK-8 and EdU assays displayed that proliferation of CAL-27 and SCC-9 cells was remarkably attenuated in the sh-PRMT5 group, in comparison to the sh-NC group (Figures 7(c) and 7(d)). Furthermore, sh-PRMT5 markedly attenuated the invasion and migration of CAL-27 and SCC-9 cells (Figures 7(e) and 7(f)).

### 3.6. METTL3 Promotes OSCC Progression through PRMT5 and PD-L1

**Figure 8:**
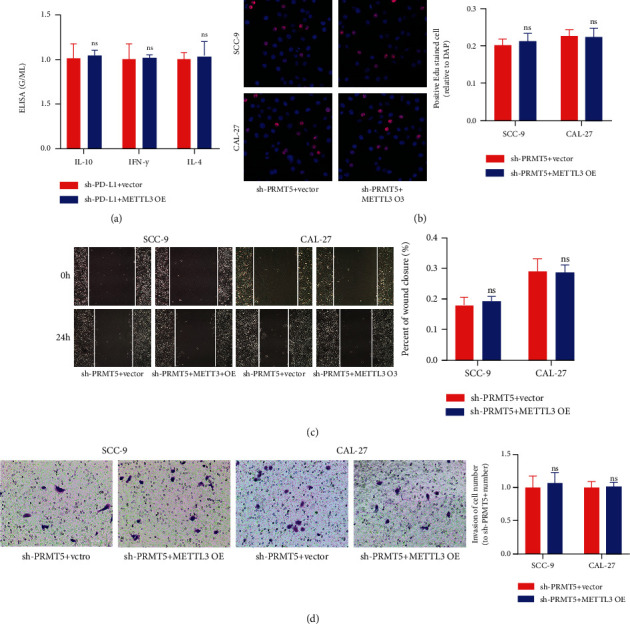
METTL3 promotes OSCC progression through PRMT5 and PD-L1. (a) METTL3 OE could not reverse or weaken the effect of sh-PD-L1 on the immune activity of CD8+ T cells. (b) EdU assay displayed that overexpression of METTL3 could not eliminate or weaken the inhibition of sh-PRMT5 on cell proliferation. (c) Overexpression of METTL3 could not eliminate or weaken the inhibition of sh-PRMT5 on cell migration. (d) Overexpression of METTL3 could not eliminate or weaken the inhibition of sh-PRMT5 on cell invasion. ns: no statistical significance.

For furtherly determining whether METTL3 expression accounts for the function of PRMT5 and PD-L1, we attempted to use METTL3 OE to reverse the effects of sh-PD-L1 and sh-PRMT5 on cells. As shown in Figure 8(a), there were no differences in IFN-*γ*, IL-4, and IL-10 between the sh-PD-L1+vector group and the sh-PD-L1+METTL3 OE group. The cotransfection of METTL3 OE could not weaken the effect of sh-PD-L1 on CD8+ T cells. Similarly, as shown in Figures 8(b)–8(d), the migration, invasion, and proliferation of OSCC cells displayed no difference between the sh-PRMT5+vector group and the sh-PRMT5+METTL3 OE group. METTL3 OE could not reverse or weaken the inhibitory effects of sh-PRMT5 on proliferation, migration, and invasion of OSCC cells. These findings demonstrated that METTL3 promotes OSCC progression by regulating PRMT5 and PD-L1.

## 4. Discussion

m6A, the most abundant modification in mRNA, is modulated by the m6A demethylases, methyltransferases, and readers. Growing evidence has confirmed that the m6A modification has an association with invasion, proliferation, tumorigenesis, and metastasis and serves as an oncogene or antioncogene in malignant tumors [15]. Therefore, we want to study OSCC from the direction of m6A, hoping to make new progress in the diagnosis or treatment of OSCC.

In our study, bioanalysis displayed that METTL3 has close relevance with the prognosis of squamous cell carcinoma of head and neck. qRT-PCR displayed that METTL3 is highly expressed in OSCC cells, and we concluded that METTL3 may play a role as an oncogene in OSCC. Subsequently, through a series of functional experiments, we found that highly expressed METTL3 could promote the progression of OSCC and inhibit the activity of CD8+ T cells, suggesting that METTL3 plays an important biological function in the development and occurrence of OSCC. Then, in order to further explore the molecular mechanism of METTL3 in OSCC, we found a good positive correlation between PRMT5 and METTL3 expressions through database analysis. In OSCC patients, PRMT5 expression was not only remarkably elevated in OSCC but also related to poor prognosis. Moreover, countless studies have demonstrated that PRMT5 promotes tumor progression. For example, through the FBW7/CMYC axis, PRMT5 intensifies glycolysis and tumorigenicity of pancreatic cancer [26]. The PRMT5/WNT4 axis intensifies proliferation and lymph node metastasis of laryngeal carcinoma [27]. PRMT5 inhibits the expression of BTG2 in hepatocellular carcinoma through the ERK signaling pathway, thereby promoting cell proliferation [28]. Besides, some researches displayed that PRMT5 expression is increased in OSCC tissues and is related to EMT [29]. However, its role in the OSCC needs to be further verified. In this study, our experiment also demonstrated that overexpression of PRMT5 can promote the invasion, migration, and proliferation of OSCC tumor cells.

METTL3 is one of the most important proteins in the m6A methyltransferase complex, which is mainly responsible for the m6A modification of RNA molecules on N6-methyladenine [22, 23]. m6A is a widely existing base modification behavior on mRNA, which can maintain mRNA stability [30]. Methylation in the 5′UTR region of mRNA plays an important part in mRNA degradation, stability, editing, splicing, polyadenylation, etc. Methylation in the 3′UTR region makes contribution to exonuclear transport of mRNA, structural stability of mRNA with polyA-binding proteins, and initiation of translation [31–33]. We hypothesized that METTL3 may mediate the methylation of PRMT5 mRNA and participate in the pathogenesis of OSCC. And in the process of experiment, we found that METTL3 can influence the activity of CD8+ T cells to kill cancer cells. At present, the PD1/PD-L1 pathway is a hotspot of tumor immunity, and we confirmed by the SRAMP database that the retrieval of PRMT5 and PD-L1 can indeed modify m6A methylation in multiple sites, so we can speculate that METTL3 by methylation modification regulates the expression of PRMT5 and PD-L1. Subsequently, through western blot, qRT-PCR, and methylation detection, we found that downregulation of METTL3 could reduce the m6A amounts of PRMT5 and PD-L1 and then reduce the expressions of PRMT5 and PD-L1. And by the torsion test, we found that METTL3 OE could not reverse the effect of sh-PD-L1 on T cell activity. At the same time, METTL3 OE could not reverse the inhibitory effect of sh-PRMT5 on OSCC cells. This suggests that METTL3 is involved in the pathogenesis of OSCC by mediating the methylation of PRMT5 and PD-L1.

In summary, METTL3 is abnormally highly expressed in OSCC. METTL3 can regulate the expression of PRMT5 and PD-L1 through methylation modification, thereby regulating the migration, proliferation, invasion, and cellular immunity of OSCC, and it may become a new target for the diagnosis and treatment of OSCC.

## Data Availability

The data used to support the findings of this study are included within the article.
